# Epigenetic association with environmental risk factors for mental disorders

**DOI:** 10.1192/j.eurpsy.2021.119

**Published:** 2021-08-13

**Authors:** S. Le Hellard

**Affiliations:** Clinical Science, University of Bergen, Bergen, Norway

## Abstract

**Abstract Body:**

Major mental disorders have typically a complex aetiology where both genetic and environmental risk factors have been implicated. It has also been suggested that these risk factors could be interactive rather than just additional. In the last decade, large genetic studies have began to unravel the genetic architecture of several of these disorders. While the mechanisms of action of environmental risk factors are still unclear. At the molecular level, gene expression can be regulated at the epigenetic level, e.g. chromatin modifications or DNA methylation. Epigenetic modifications can be affected by both genetic variations as well as environment variations. In this presentation, we will review recent results either from literature or from own data on how several known environmental risks for mental disorders can be associated with modifications of epigenetic markers, especially in DNA methylation. We will for instance look at the modifications associated with smoking, alcohol, cannabis, childhood trauma or obstetric complications. We will discuss also the limits of these studies and how epigenetic modifications can be relevant for the onset of mental disorders and their treatment.

**Disclosure:**

No significant relationships.

